# Inhibition of Epac2 Attenuates Neural Cell Apoptosis and Improves Neurological Deficits in a Rat Model of Traumatic Brain Injury

**DOI:** 10.3389/fnins.2018.00263

**Published:** 2018-04-23

**Authors:** Ling Zhang, Li Zhang, Huixiang Liu, Feng Jiang, Huanjing Wang, Di Li, Rong Gao

**Affiliations:** ^1^Translational Medicine Center, The First People's Hospital of Zhangjiagang, Zhangjiagang, China; ^2^Department of Neurosurgery, The First People's Hospital of Zhangjiagang, Zhangjiagang, China; ^3^Department of Neurosurgery, Zhangjiagang Hospital of Traditional Chinese Medicine, Zhangjiagang, China

**Keywords:** traumatic brain injury, Epac2, P38, apoptosis, neuroprotection

## Abstract

Traumatic brain injury (TBI) is a major cause of mortality and disability worldwide. TBI-induced neuronal apoptosis is one of the main contributors to the secondary injury process. The aim of this study is to investigate the involvement of Exchange protein directly activated by cAMP 2 (Epac2) on TBI. We found that the expression level of Epac2 surrounding the injured area of brain in rats of TBI model was significantly increased at 12 h after TBI. The role of Epac2 in TBI was further explored by using a selective Epac2 antagonist ESI-05 to decrease the Epac2 expression. We discovered that inhibition of Epac2 could improve the neurological impairment and attenuate brain edema following TBI. The Epac2 inhibition effectively reduced neuronal cell death and P38 MAPK signaling pathway may be involved in this process. Our results suggest that inhibition of Epac2 may be a potential therapy for TBI by reducing the neural cell death, alleviating brain edema and improving neurologic deficits.

## Introduction

Traumatic brain injury (TBI) is one of the leading causes of mortality and disability all over the world (Menon and Maas, 2015; Wang et al., 2018). TBI can result in physical, cognitive, social, emotional, and behavioral symptoms. TBI consists of a primary mechanical brain tissue injury that occur at the time of the initial trauma and a secondary insult with a series of pathological responses, including intracerebral hemorrhage, oxidative stress, neuroinflammation, blood-brain barrier (BBB) damage, autophage, and apoptosis (Cornelius et al., 2013; Li et al., 2017; Tang et al., 2017). The long-term consequence of TBI was dominated by the secondary injury, so the secondary brain injury has been the major focus to identify potential therapeutic targets in TBI management.

Epac (Exchange protein directly activated by cAMP) proteins have two isoforms, Epac1 and Epac2 (Gloerich and Bos, 2010). Epac1 is expressed throughout the body, while Epac2, the larger of the two isoforms, is highly enriched in brain and adrenals (Kawasaki et al., 1998). Epac2 is a guanine nucleotide exchange factor (GEF) to activate the small GTPase Rap (Kawasaki et al., 1998; Bos, 2003). Deletion of Epac2 causes brain dysfunction, such as impairments in memory and social interaction (Fernandes et al., 2015; Lee et al., 2015). Epac2 activation was associated with astrocytic differentiation and inflammation (Oldenburger et al., 2014; Seo and Lee, 2016). Epac2 promotes neurotransmission in the hippocampus (Fernandes et al., 2015). Epac2 has been proposed as a promising target for treatment of diabetes, cancer and cardiovascular disease (Zhang et al., 2009; Parnell et al., 2015; Yang et al., 2017). However, the roles of Epac2 in traumatic brain injury are still unknown. In this study, we investigated the effects of Epac2 on neurological damage of TBI rat models, and demonstrated the possible signaling pathway involved in this process.

## Materials and methods

### Animals

Male Sprague-Dawley rats (280–300 g) were obtained from the Animal Center of Chinese Academy of Sciences, Shanghai, China. The animals were housed under controlled environmental conditions with a 12 h light/dark cycle place and were given unrestricted access to pellet food and water throughout the study. All experimental procedures were approved by the Institutional Animal Care and Use Committee of Soochow University and conformed to the National Institutes of Health Guide for the Care and Use of Laboratory Animals.

### TBI model

**Figure 1:**
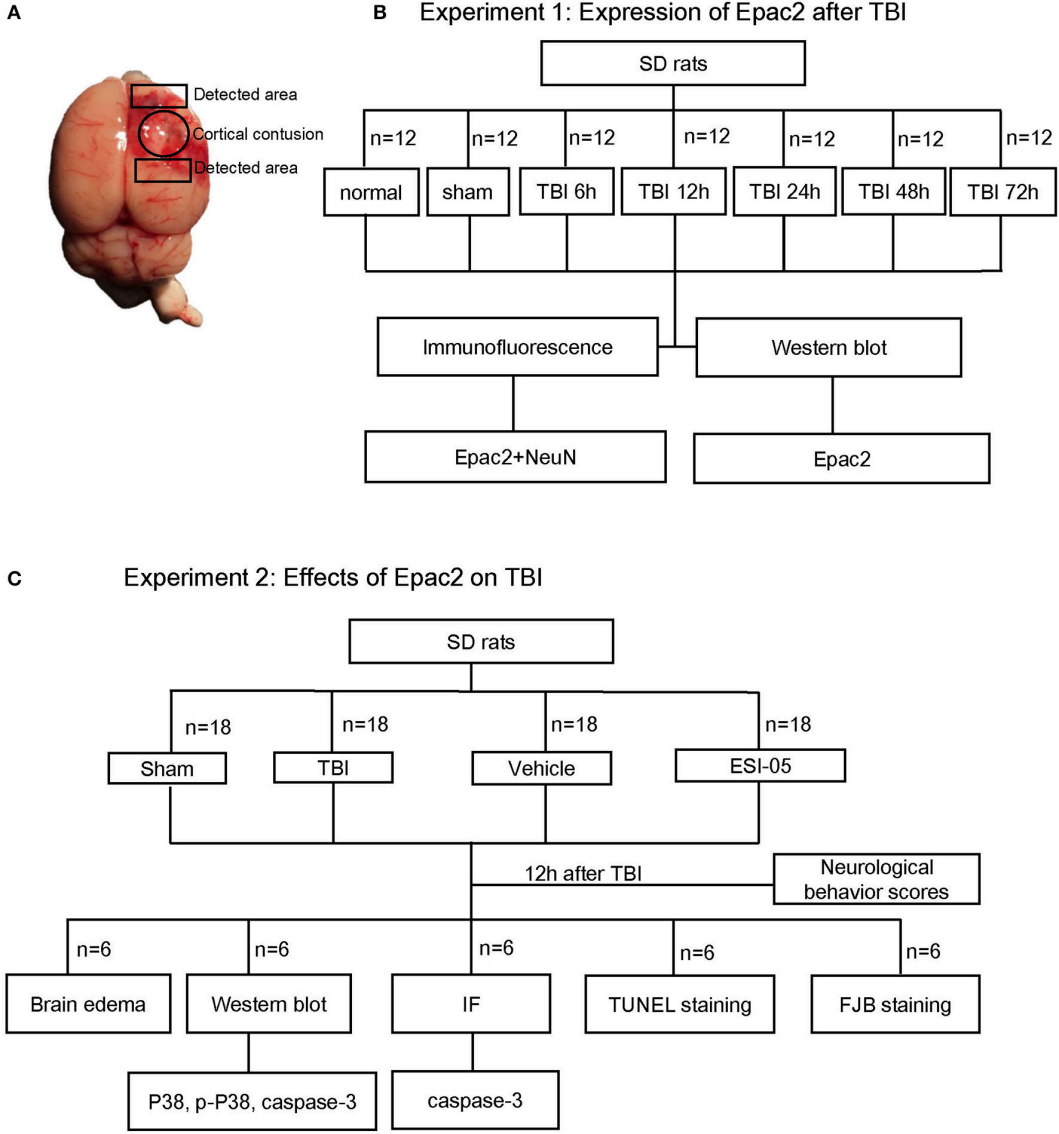
Schematic representation of TBI model and experimental design. **(A)** Brains of TBI model, the studied region is surrounding the injured brain. **(B)** Experiment 1 was designed to detect the time course of Epac2 expression after TBI. **(C)** Experiment 2 was designed to detect the effects of Epac2 on TBI and investigate the possible mechanism.

Surgical procedures were performed as previously described (Li et al., 2017). The rats were anesthetized by an intraperitoneal injection of 4% chloral hydrate (10 ml/kg) (Dang et al., 2015). The head of them was fixed on a stereotaxic frame. After incision of the scalp, a craniotomy of 5 mm diameter was performed beside midline and behind the cranial coronal suture using a dental drill. Contusion was produced by letting a 40 g weight cylindrical steel rod (diameter 4 mm) drop onto the piston resting on the exposed dura from a height of 25 cm. The piston was allowed to compress the tissue a maximum of 5 mm (Hang et al., 2004). Then the scalp was sutured and the wound area was treated with lidocaine cream. In the sham group, animals underwent identical procedures, including craniotomy, but without brain injury. As shown in Figure 1A, the surrounding brain tissue of the injured cortex was dissected on ice, some of which were placed in 4% PFA, the others were stored in liquid nitrogen immediately until use.

### Experimental design

There was no significant difference in body temperature, weight, feed intake, and motor ability of all rats before the experiment.

1. Experiment 1. To explore the expression levels of Epac2 at each time point after TBI, SD rats were randomly assigned into three groups: the normal group (*n* = 12), the sham group (*n* = 12), and the TBI group (*n* = 60). The TBI group were divided into five subgroups (*n* = 12 for each time point) at 6, 12, 24, 48, and 72 h after TBI, respectively. All rats were sacrificed at the planned time point and the cortical tissue samples were collected for subsequent analysis such as western blot and immunofluorescence staining (Figure 1B). Mortality in the normal group and sham group is 0%, while in TBI group is 12% (8 of 68).
2. Experiment 2. To investigate the effects of Epac2 on TBI, we used the Epac2 selective antagonist ESI-05 (Sigma-Aldrich, SML1907) to reduce the expression level of Epac2 (Rehmann, 2013). Then we detect the changes of neuronal apoptosis, BBB damage, brain edema and other indicators after TBI. SD rats were randomly assigned into four groups: the sham group (*n* = 18), the TBI group (*n* = 18), the vehicle group (*n* = 18), and the ESI-05 group (*n* = 18). The vehicle group and the ESI-05 group were respectively injected into lateral ventricles with 1% DMSO (15 μl) and ESI-05 (10 μg/kg, dissolved in 1% DMSO) 30 min before TBI. At 12 h after TBI, all rats were sacrificed and samples were collected for subsequent analysis (Figure 1C). Mortality in the sham group is 0% (0 of 18), in TBI group is 14% (3 of 21), in vehicle group is 18% (4 of 22), and in ESI-05 group is 14% (3 of 21).

### Western blot

The cortical regions of the brains were collected and homogenized in the lysis buffer containing protease inhibitor. The proteins were extracted and the protein concentration was measured using a BCA protein assay kit (Thermo, 23227). Equal quantities of protein (40 μg) from each samples were loaded for SDS-PAGE. After electrophoresis, the protein was transferred onto polyvinylidene difluoride membranes (GE Healthcare, RPN303F). The membranes were blocked with 5% nonfat milk for 1 h at room temperature and subsequently incubated overnight at 4°C with the following primary antibodies: Epac2 (1:1,000, Cell Signaling, 43239), P38 (1:1,000, Abcam, ab17009), P-P38 (1:500, Abcam, ab38238), caspase-3 (1:1,000, Abcam, ab13847), GAPDH (1:10,000, Sigma, G9545). The membranes were incubated with appropriate secondary antibodies for 2 h at room temperature. The target band signals were detected by Enhanced chemiluminescence (ECL) method. The signals were quantified using Image J software.

### Immunofluorescence staining

The rats were sacrificed at 12 h after TBI. The brain tissue samples were immersed in the 4% paraformaldehyde for 36 h at 4°C and then gradient dehydrated with 15 and 30% sucrose solution until they sank to the bottom. Then frozen sections with a thickness of 15 μm were collected. The brain sections were rinsed in phosphate-buffered saline (PBS) with 0.3% Triton X-100 for 10 min, repeat 3 times. The sections were incubated with 10% normal horse serum for 1 h at room temperature to prevent nonspecific binding. Then the sections were incubated at 4°C overnight with the following primary antibodies: NeuN (Millipore, MAB377, 1:300), Epac2 (1:200, Cell Signaling, 43239), caspase-3 (1:200, Abcam, ab13847). After washing for 3 times with PBS, the sections were incubated with the fluorescent secondary antibodies for 1 h at room temperature in the dark. After washing for 3 times, the sections were covered with DAPI Fluoromount-G. Immunofluorescence staining was observed using a laser confocal microscopy (Leica, TCS SP8).

### FJB staining

Fluoro Jade B (FJB) (Histo-Chem, Jefferson) staining was used for detection of the damaged neurons. The sections were treated with 1% sodium hydroxide in 80% alcohol for 5 min, 70% alcohol for 2 min and rinsed with ddH2O for 2 min. The sections were then immersed in a solution of 0.06% potassium permanganate for 10 min and rinsed with ddH2O for 2 min. Subsequently, the sections were incubated in FJB staining solution (0.001% FJB in 0.1% acetic acid) for 20 min at room temperature and rinsed three times with ddH2O. The sections were dried at 50°C for about 5–10 min. Then the slides were placed in xylene for 1 min before mounted with resinene.

### Tunnel staining

A TUNEL staining kit (Abcam, ab66110) was used to analyze apoptotic cell death. Frozen sections were fixed with fresh 4% paraformaldehyde for 15 min at room temperature. After washing with PBS for 2 times, the sections were covered with 20 μg/ml Proteinase K solution for each one and incubated for 5 min at room temperature. Then the sections were covered with 100 μl wash buffer and incubated at RT for 5 min. Then 50 μl DNA labeling solution was covered on the sections. Place the slides in a dark humidified incubator for 1 h at 37°C. After washing with ddH2O, the sections were incubated for 5 min at RT. Cover the sections with DAPI Fluoromount-G and observe under a laser confocal microscopy (Leica, TCS SP8).

### Neurological evaluation

Neurological function was evaluated in all rats of each group in Experiment 2 at 12 h after TBI. The appetite, activity, and neurological deficits of rats were assessed according to the previously published scoring system (Table 1). The highest score of 9 represents the most severe neurological deficit, while the lowest score of 0 indicates normal neurological function.

**Table 1:** Assessment of neurological behavior scores.

**Category**	**Behavior**	**Score**
Appetite	Finished meal	0
	Left meal unfinished	1
	Scarcely eat	2
Activity	Move freely in the cage	0
	Move under stimulus	1
	Barely moved	2
Deficits	No deficits	0
	Walk unsteadily	1
	Unable to walk	2

### Brain water content measurement

Six rats in each group of Experiment 2 were used for measurement of brain water content. Rats were sacrificed after 12 h of TBI. The brains were removed and the right hemispheres were collected. Immediately weigh the samples and the weight was recorded as wet weight. The samples were then dried at 100°C for 24 h and the weight at the moment was recorded as dry weight. The brain water content was calculated using the formula (wet weight-dry weight)/wet weight × 100%.

### Statistical analysis

To facilitate comparisons between groups, the western blot results were expressed as relative density of the band as compared GAPDH and then normalized to the mean value of the sham group. All data in this paper are presented as mean ± SE. SPSS Statistics was used for statistical analysis. The results were analyzed using one-way ANOVA test followed by Bonferroni's multiple comparison test. Statistical significance was accepted at *P* < 0.05.

## Results

### The expression of Epac2 was increased after TBI

**Figure 2:**
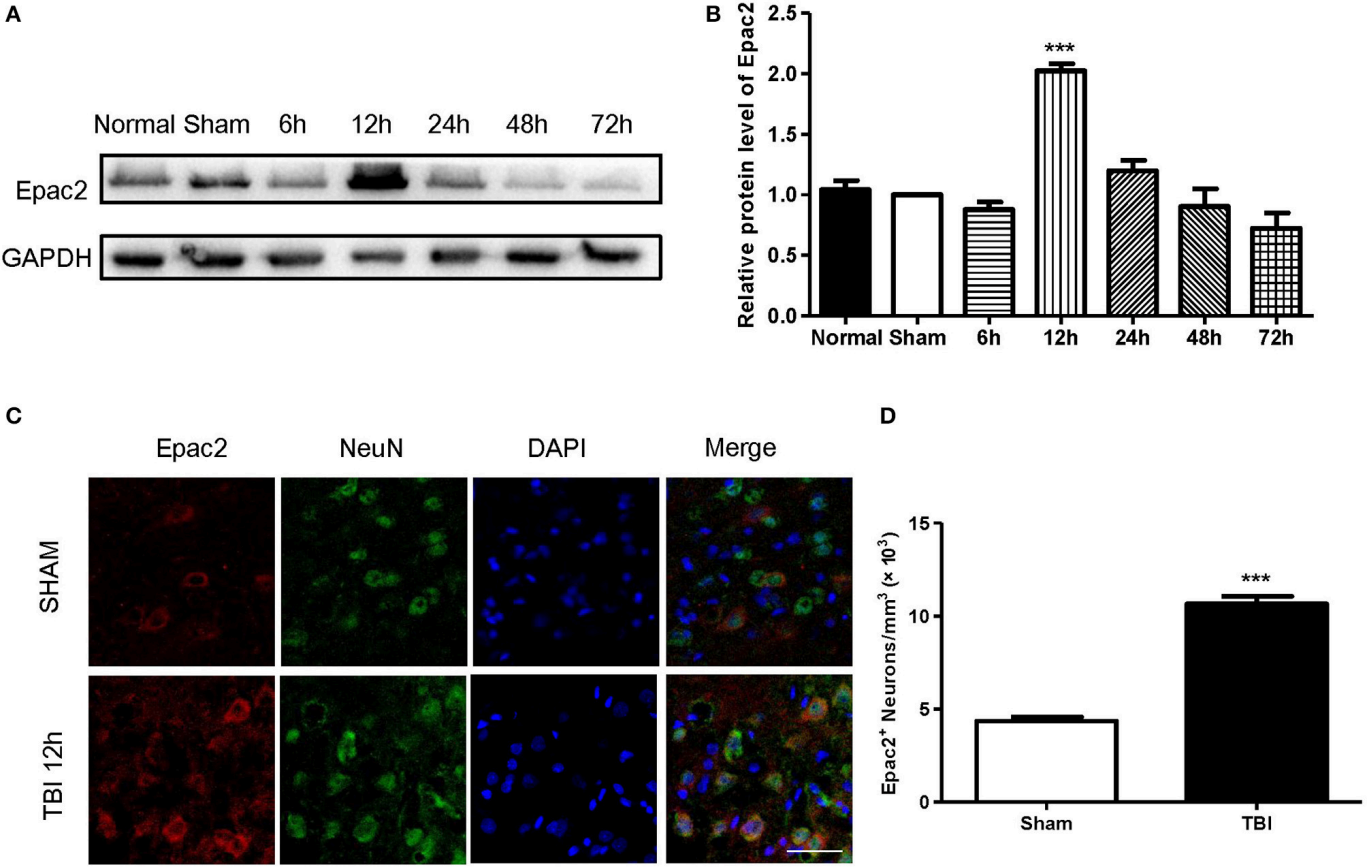
Epac2 was co-located with neurons and dramatically increased at 12 h after TBI. **(A)** Western blot analysis showed the expression of Epac2 in normal and sham group and at 6, 12, 24, 48, and 72 h after TBI. GAPDH was used as a loading control. **(B)** Quantification of western blots for Epac2 as shown in **(A)**. **(C)** Immunofluorescence staining result showed that Epac2 (red) was expressed in neurons (green) and the expression level of Epac2 was increased at 12 h after TBI. **(D)** Quantitative analysis of Epac2-positive neurons. Scale bar = 50 μm. Data are presented as means ± SE. *n* = 6 in each group. ***P < 0.001 vs. sham group.

To investigate the possible participation of Epac2 in the pathogenesis of TBI, we examined the expression and localization of Epac2 in brains subjected to TBI. Western blot was used to explore the expression level of Epac2 at different time point after TBI. The results showed that the expression level of Epac2 was dramatically increased at 12 h after TBI (Figures 2A,B). We used immunofluorescence staining to detect the expression and localization of Epac2 in brain. The results showed that Epac2 was colocalized with neuron and conformed that Epac2 expression level was significantly elevated in TBI model rats (Figures 2C,D).

### The decrease of Epac2 alleviated neurological deficits following TBI

**Figure 3:**
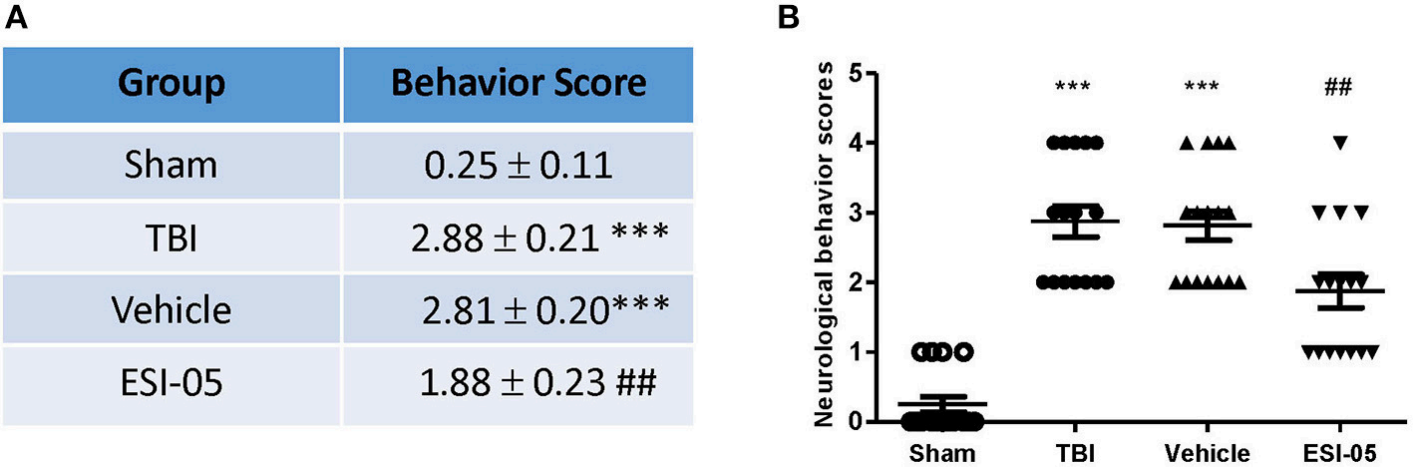
Decrease of Epac2 improved neurological behavior impairment following TBI. **(A)** A table showed the neurological behavior scores in each group. **(B)** Scatter plot showed the neurological behavior scores of all rats in each group. Data are presented as means ± SE. *n* = 18 in each group. ***P < 0.001 vs. sham group, ^##^*P* < 0.01 vs. vehicle group.

Previous studies revealed that the neurological functions were impaired in TBI model. To assess whether Epac2 contributes to TBI, we used a loss-of-function strategy to evaluate the effect of Epac2 inhibition on behavioral recovery. The experimental results showed that the neurological behavior scores were significantly higher than the sham group, indicating that the rats of TBI group had a significant neurological function deficits compared with sham group. ESI-05 treatment could significantly decrease the neurological behavior scores (Figures 3A,B). These findings suggest that Epac2 inhibition improved neurological behavioral impairment following TBI. Decrease of Epac2 expression level contributed to alleviate neurological deficits after TBI.

### The reduction of Epac2 decreased brain water content after TBI

**Figure 4:**
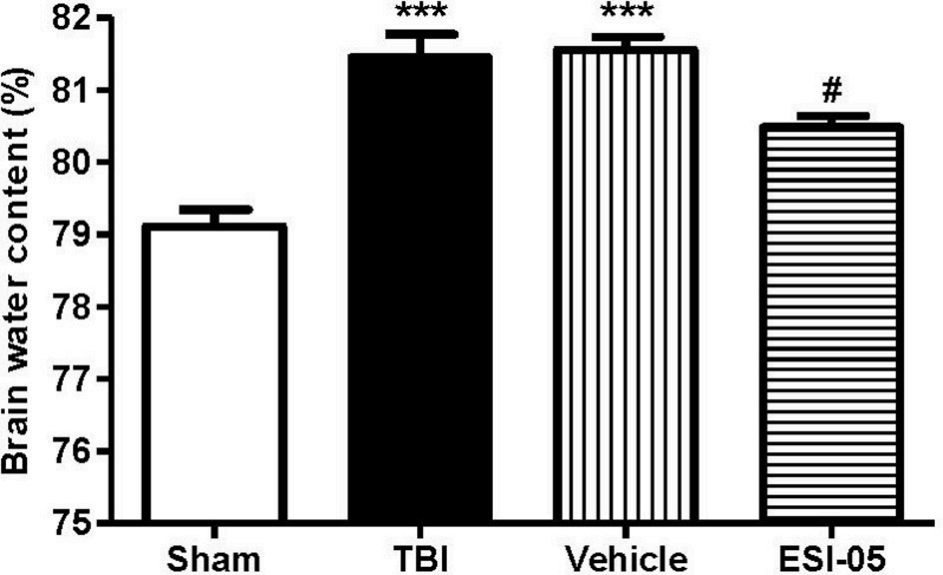
Reduction of Epac2 alleviated brain edema after TBI. Brain water content of cerebrum in each group was measured by dry/wet method. Data are presented as means ± SE. *n* = 6 in each group. ***P < 0.001 vs. sham group, ^#^*P* < 0.05 vs. vehicle group.

The expression of Epac2 was significantly increased at 12 h after TBI in Experiment 1. In order to explore the effects of Epac2 in pathological progress of TBI, in Experiment 2, we used ESI-05, the specific antagonist of Epac2, to decrease the Epac2 expression level. Brain water content represented the severity of brain edema. We compared the brain water content in the sham group, TBI group, vehicle group and ESI-05 group after TBI. We found that the brain water content was significantly increased in TBI model rats, and ESI-05 treatment could significantly reduce it while vehicle group couldn't (Figure 4).

### Inhibition of Epac2 attenuated neural cell apoptosis after TBI

**Figure 5:**
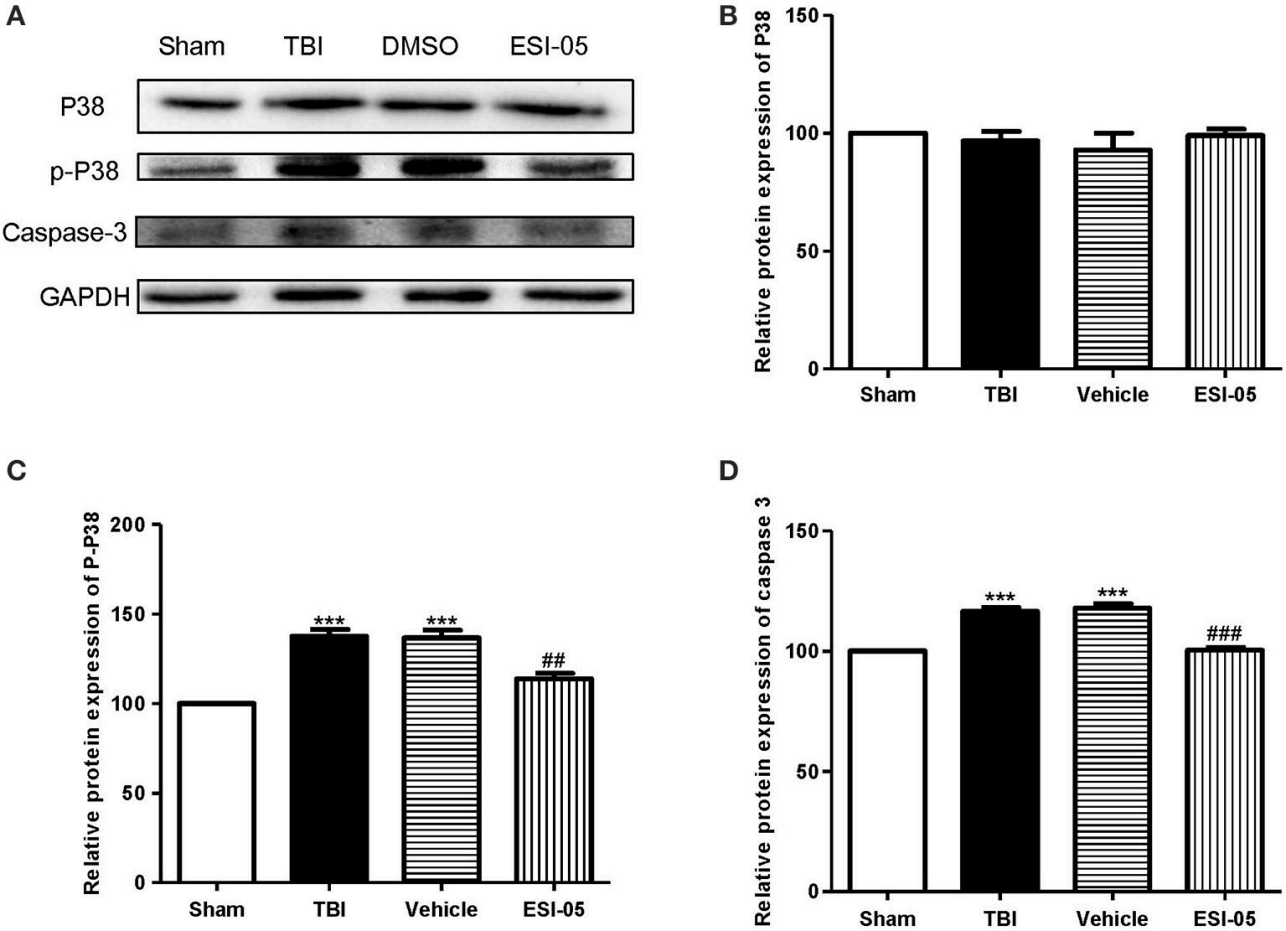
Inhibition of Epac2 reduced apoptosis related protein caspase-3 in brain after TBI through inhibiting phosphorylation of P38. **(A)** Western blot analysis showed the expression of P38, p-P38, and caspase-3 in cerebral cortex in each group. GAPDH was used as a loading control. **(B–D)** Quantification of expression levels of P38, p-P38, and caspase-3 as shown in **(A)**. Data are presented as means ± SE. *n* = 6 in each group. ***P < 0.001 vs. sham group, ^##^*P* < 0.01, ^###^*P* < 0.001 vs. vehicle group.

**Figure 6:**
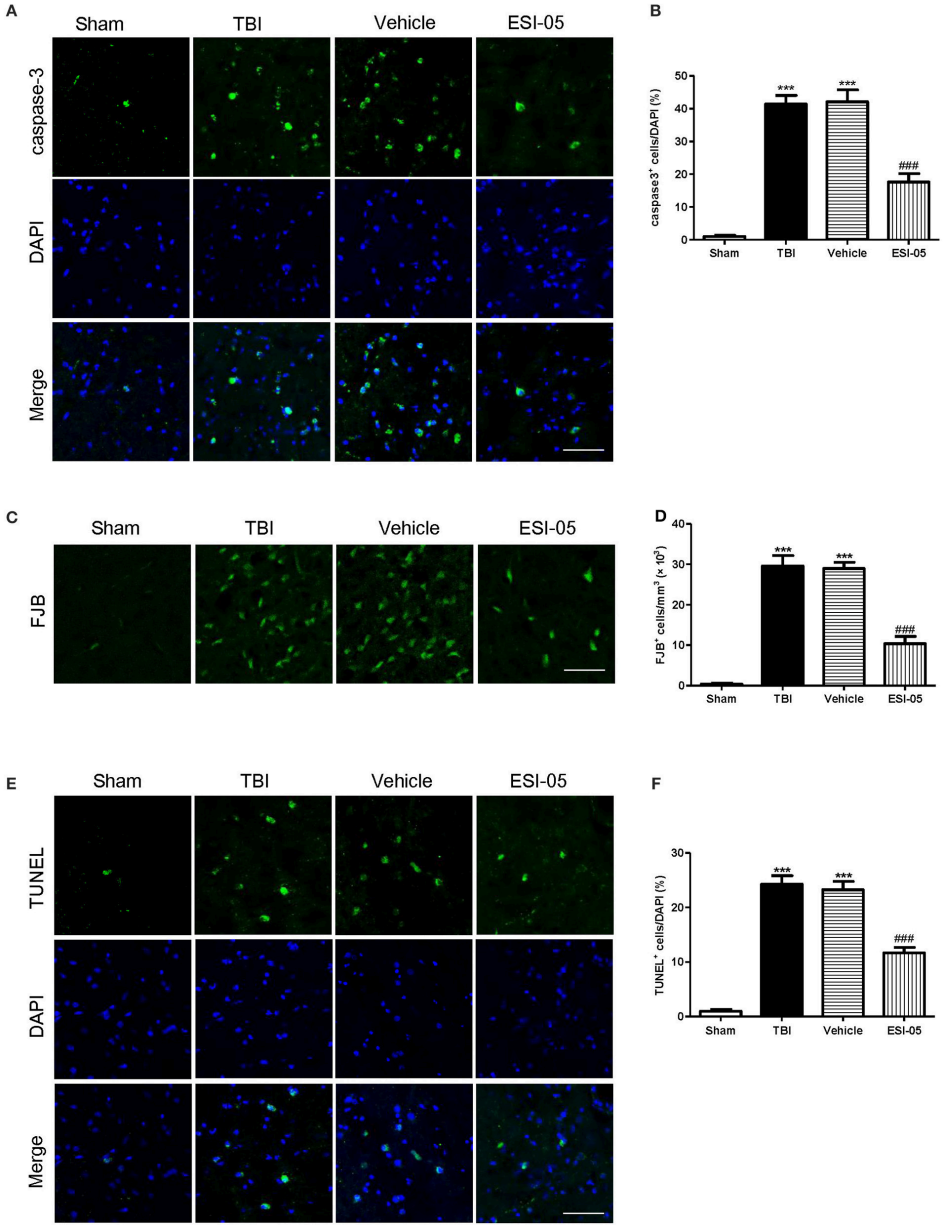
Inhibition of Epac2 attenuated neural cell death in cerebral cortex after TBI. **(A,C,E)** Cortical cellular apoptosis was detected by immunofluorescence staining of caspase-3 (green), FJB staining (green), and TUNEL (green) staining in each group, respectively. **(B,D,F)** Quantitative analysis of caspase-3, FJB, and TUNEL-positive cells in cerebral cortex after TBI, respectively. Scale bar = 50 μm. Data are presented as means ± SE. *n* = 6 in each group. ***P < 0.001 vs. sham group, ^###^*P* < 0.001 vs. vehicle group.

The neuron apoptosis has always been the focus in studies of brain injury (Xu et al., 2016; Tang et al., 2017). We performed immunofluorescence staining and western blot of caspase-3, FJB staining and TUNEL staining to explore the role of Epac2 in neural cell death. Both of the western blot and immunofluorescence staining results showed that the expression of caspase-3 was significantly decreased in the ESI-05 treated group (Figures 5A,D, 6A,B). Few FJB-positive and TUNEL-positive apoptotic cells was found in the sham group. And the number of apoptotic cells was dramatically higher in TBI group. The FJB and TUNEL staining results also demonstrated that inhibition of Epac2 could significantly prevent the increase of cell death (Figures 6C–F). As shown in Figure 5, the expression of P38 has no significance between each experimental groups (Figure 5B). But the expression of p-P38 was markedly increased after TBI and treatment with ESI-05 could reverse this change (Figure 5C). These results indicated that inhibition of Epac2 could reduce TBI-induce neural cell apoptosis and the inhibition of phosphorylation of P38 MAPK pathway may be involved.

## Discussion

The pathological changes including inflammation, oxidative damage, apoptosis and brain edema are the main causes of the secondary brain injury after TBI (Jennings et al., 2008; Onyszchuk et al., 2008). The key to treatment of TBI had to focus on how to alleviate the secondary damage after TBI. In this study, we explored the regulation of Epac2 in the secondary brain damage after TBI and studied the potential mechanisms. We found that the treatment of the selective Epac2 antagonist ESI-05 reduced neural cell death in the injured cerebral cortex and adjacent regions in rats of TBI models. Inhibition of Epac2 decreased TBI-induced P38 phosphorylation and caspase-3 activation in the cerebral cortex, attenuated neural cell death, alleviated brain edema and improved neurological functions after TBI.

Increased apoptosis takes an important part in the pathogenesis of brain damage. Caspases are a family of cysteine proteases which play an important role in apoptosis. Caspase-3, as a key molecule of apoptosis, plays a central role in the neural cell apoptosis after TBI (Clark et al., 2000). Our findings revealed a significant increase in cleaved caspase-3 after TBI, suggesting an increase of apoptosis in the brain following TBI. FJB, and TUNEL staining also confirmed increase of neural cell death after TBI. However, ESI-05 treatment ameliorated this TBI-induced neural cell apoptosis, indicating that the decrease of Epac2 could inhibit the TBI-induced neural cell death. This result was consistent with previous study showing that Epac2 was involved in apoptosis (Park and Juhnn, 2017).

Numerous studies have suggested the relationship between Epac2 and cell death. Previous studies showed that Urocortion-1 promoted cell survival and decreased cell necrosis through Epac2 and ERK1/2 (extracellular signal-regulated kinases 1/2) activation (Calderón-Sánchez et al., 2016). In H1299 lung cancer cells, Epac2 inhibition decreased cisplatin-induced apoptosis via Epac2-Rap1-Akt pathway (Park and Juhnn, 2017). Epac2 mediates cAMP-dependent growth arrest via activating Rap2A in neuroendocrine cells (Emery et al., 2017). Epac2-Rap1 signaling also attenuates mitochondrial ROS production and reduces myocardial arrhythmia susceptibility (Yang et al., 2017). In rat model of plantar incision, Epac2 mediates nociception priming through P38 pathway (Matsuda et al., 2017). In this study we explored the mechanisms of Epac2 mediates apoptosis in brain following TBI.

**Figure 7:**
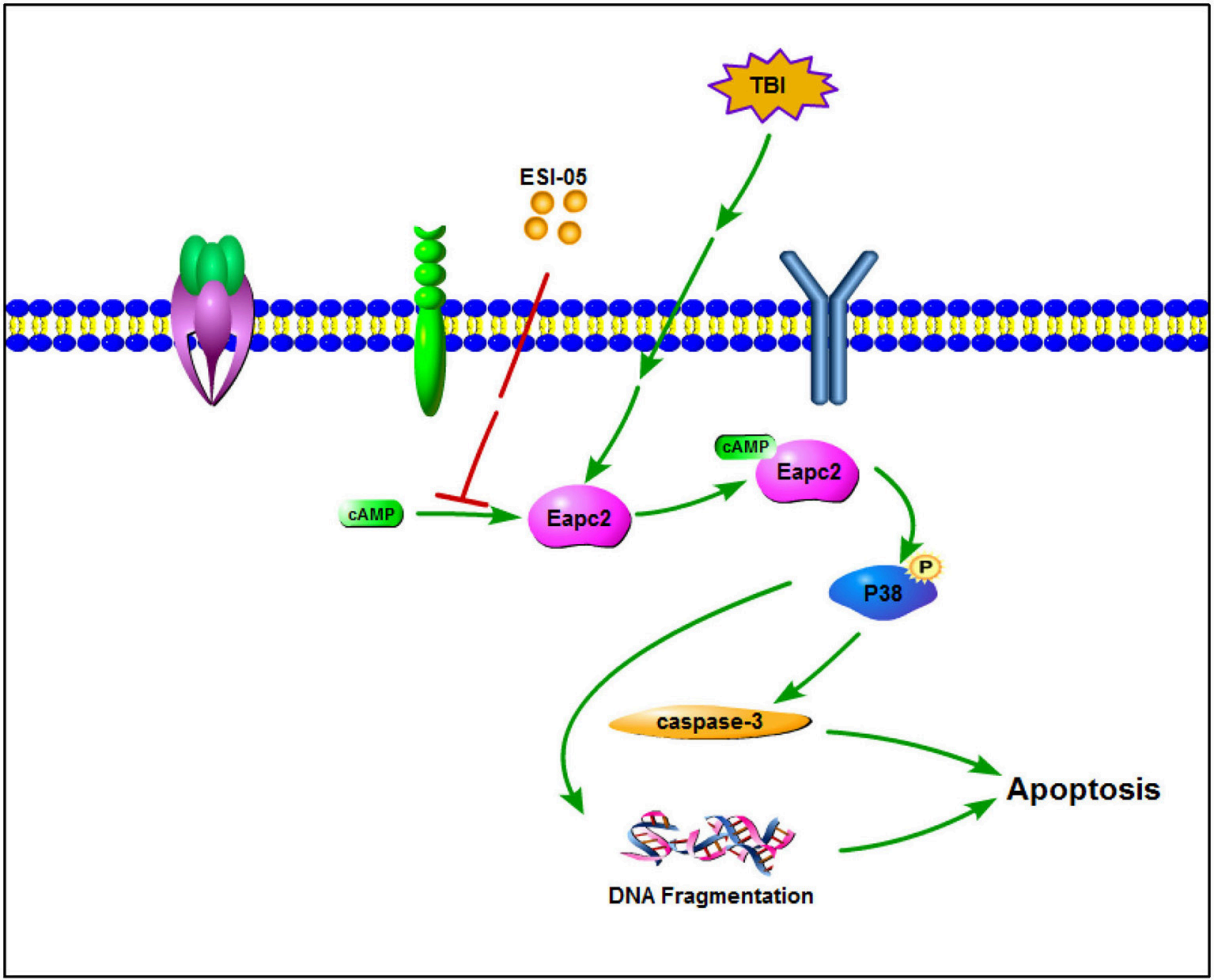
A schematic model for Eapc2 regulation of neuronal cell death in TBI. Epac2 was increased after TBI and activating Epac2 subsequently promoted phosphorylation of P38-MAPK. Activation of P38-MAPK pathway consequently increased the expression of caspase-3, resulted in DNA fragmentation and neuron apoptosis, leading to neuronal cell death.

Mitogen-activated protein kinases (MAPKs) are serine/threonine protein kinases. They regulate cell functions including proliferation, differentiation, stress response, mitosis, cell survival and apoptosis (Pearson et al., 2001). P38 MAPK is one of the well-characterized MAPK family members. Increasing data has shown that activated P38 MAPK subsequently regulates the inflammatory response, apoptosis, autophagy, cell survival, and cell death (Xia et al., 1995; Harada and Sugimoto, 1999; Nozaki et al., 2001; Irving and Bamford, 2002; Sridharan et al., 2011; Sui et al., 2014). Several studies showed that increased P38 MAPK activity plays a key role in neural cell death and inhibition of p38 MAPK plays a neuroprotective role (Takeda and Ichijo, 2002; Strassburger et al., 2008; Liu et al., 2014; Li et al., 2015; Wang et al., 2015). Epac proteins have been characterized as guanine nucleotide exchange factors for monomeric GTPases like Rap1 (Kawasaki et al., 1998) and Rab3a (Branham et al., 2009). The relationship between Rap1 and P38 has been shown in several studies (Gutiérrez-Uzquiza et al., 2010; Wu et al., 2015; Lu et al., 2016; Priego et al., 2016). In the present study, we also found that phosphorylation level of P38 protein was increased after TBI. Treatment with ESI-05 decreased this level. These results demonstrated that P38 MAPK pathway may involve in the regulation of neuronal apoptosis after TBI. Inhibition of Epac2 plays an anti-apoptotic role in TBI model rats and this process may be mediated through regulating P38 MAPK pathway (Figure 7). These results tallied with previous studies demonstrating that Epac2 could mediate growth arrest through P38 activation (Emery et al., 2017). To further conform the involvement of the P38 MAPK signaling pathway in Epac2's effect on TBI, SB203580, an inhibitor of P38 phosphorylation is needed to be used in the further investigation.

Cerebral edema is a dangerous secondary consequence of TBI and is associated with significant morbidity and mortality (Winkler et al., 2016). The development of cerebral edema plays a crucial role in the evolution of injury following TBI. Even a minor increase of brain water content can lead to a significant increase of intracranial pressure and the poor outcome (Marmarou et al., 2000). Thus alleviate brain edema is a promising treatment in TBI management. In the present study, we found that reduction of Epac2 could significantly alleviated brain water content of the injured hemisphere. This result might be due to the alleviation of blood brain barrier disruption. TBI increases the expression of inflammation factors, chemotactic factors and adhesion molecules in the neural system, which leads to encephaledema. Epac2 may be involve in the brain edema after TBI through affecting these factors. The mechanisms Epac2 involved in these processes need to be further investigated.

## Conclusion

In summary, western blot and immunofluorescence staining results have shown that the expression of Epac2 was dramatically increased at 12 h after TBI. The brain water content measurement showed that reduction of Epac2 alleviated encephaledema in TBI model. The neurological behavioral test demonstrated that decrease of Epac2 improved neurobehavioral outcome after TBI. The immunohistochemistry, FJB, TUNEL, western blot were used to show that inhibition of Epac2 significantly attenuated the neuronal cell death after TBI. Phosphorylation of P38 was involved in this process. These data suggested that inhibition of Epac2 may play a neuroprotective role in TBI management through attenuating neural cell death, alleviating brain edema and improving neurological deficits, implying that Epac2 could be a new target for treatment of secondary neuronal injury after TBI.

## Author contributions

DL and RG designed the experiments and edited the manuscript. LingZ and LiZ performed the experiments, analyzed the data and wrote the manuscript. HL and FJ interpreted the data and prepared the figures. HW performed the experiments and analyzed the data.

### Conflict of interest statement

The authors declare that the research was conducted in the absence of any commercial or financial relationships that could be construed as a potential conflict of interest.
